# Then and Now

**Published:** 1897-08

**Authors:** 


### Then and Now.

Within fifty years wonderful changes in conditions of practice have transpired. To illustrate: Fifty years ago but a very small percentage of practicing physicians wrote prescriptions, there were no specialists in medicine, three to six chairs constituted a medical college faculty, many practitioners had never entered a medical college. Because of these general conditions, medicine as a profession was at a very low ebb, and the organization of the American Medical Association was for the express purpose of placing professional education upon a higher plane.

To this there arose an antagonism which has existed ever since, and is to be found at this very time, but to a limited extent, while within the ranks of educated physicians pessimists are to be found, and ever will be.

Since fifty years ago there has been created a practically new education in medicine. Then there was not a single State with any law upon the subject of requirements for practice; now nearly every State has a more or less stringent law, and there is not more than one which does not require a legal registration of its practitioners.

Singularly enough, Ohio was one of the last to enact a medical law of registration and examination, and this only went into effect about eight months ago, since which time the State Board has required the registration of the qualified practitioners of the State.—*Cincinnati Lancet-Clinic.*

